# How Audiences Engage With Drama: Identification, Attribution and Moral Approval

**DOI:** 10.3389/fpsyg.2021.762011

**Published:** 2021-11-05

**Authors:** Ben Teasdale, Laurie Maguire, Felix Budelmann, R. I. M. Dunbar

**Affiliations:** ^1^Calleva Research Centre, Magdalen College, Oxford, United Kingdom; ^2^Department of Experimental Psychology, University of Oxford, Oxford, United Kingdom

**Keywords:** drama, fictional transportation, identification, moral approval, attribution

## Abstract

Fictional storytelling has played an important role in human cultural life since earliest times, and we are willing to invest significant quantities of time, mental effort and money in it. Nonetheless, the psychological mechanisms that make this possible, and how they relate to the mechanisms that underpin real-world social relationships, remain understudied. We explore three factors: identification (the capacity to identify with a character), moral approval and causal attribution with respect to a character’s behaviour in live performances of two plays from the European literary canon. There were significant correlations between the extent to which subjects identified with a character and their moral approval of that character’s behaviour that was independent of the way the play was directed. However, the subjects’ psychological explanations for a character’s behaviour (attribution) were independent of whether or not they identified with, or morally approved of, the character. These data extend previous findings by showing that moral approval plays an important role in facilitating identification even in live drama. Despite being transported by an unfolding drama, audiences do not necessarily become biased in their psychological understanding of why characters behaved as they did. The psychology of drama offers significant insights into the psychological processes that underpin our everyday social world.

## Introduction

Storytelling is a uniquely human activity whose evolutionary function remains unclear, although it likely provides a means of transmitting a culture’s core beliefs and the worldview that forms an important basis for creating a sense of community (Dunbar, 2014). In contemporary hunter-gatherer societies like the Kung San, fireside conversations that involve stories predominate in the evening (Weissner, 2014). Although such stories commonly include origin stories and accounts of travels, they have probably always included fictional or semi-fictional accounts. In all cultures, fictional stories have come to play an unusually important role, providing not only a corpus of well-loved stories that define a culture but also forms of entertainment on which we are willing to spend considerable amounts of time and money. Even in traditional societies where storytellers are not paid for their efforts, people are nonetheless willing to spend considerable time being entertained by them, at no small cost in terms of potentially more functional uses of their time. Indeed, we seem to be universally willing to listen to the same well-loved stories over and over again without satiation (Leavitt and Christenfeld, 2011), with the same stories being maintained over long periods of time and very considerable geographical distances (e.g. the ‘Tale of the Two Sisters’ which appears in various forms as a folktale all over Europe: Ross et al., 2013). The magnitude of these costs, whether measured in terms of time or money, suggests that this form of activity is evolutionarily important and not some trivial by-product of a capacity designed to subserve a more important purpose.

We are so immersed in storytelling in everyday life that we forget how complex the cognitive processes of understanding and enjoying stories actually are. The most engaging stories are about *people* – or things that are accredited, for the purposes of the story, with human psychological attributes (such as talking animals, fictional beings like fairies or even trees and rocks when these are described as having minds). These include, minimally, the capacity to mentalise (in its simplest form, theory of mind) in order to be able to understand the mindstates of the storyteller and the character in the story, as well as distinguish between the real and fictional worlds (Dunbar, 2008, 2017; Carney et al., 2014), the capacity to identify with (Cohen, 2001; Tal-Or and Cohen, 2010) and empathise with (Green and Brock, 2000; Green et al., 2004) the fictional characters as portrayed, and transportation (the capacity to become immersed in a story: Green et al., 2004; Green, 2005). In this respect, of course, fictional stories necessarily depend on the same cognitive mechanisms as our capacity to tell factual stories.

Mentalising and identification (as defined by Tal-Or and Cohen, 2010: see Table 1) would seem to be defined very similarly (being able to understand the mindstate of another individual). For present purposes, we treat them as synonymous concepts. Though ostensibly similar, mentalising (and hence identification) differs from empathy in being a form of ‘cold cognition’ (beliefs about mindstates), whereas empathy is a form of ‘hot cognition’ (emotional feelings). Higher-order mentalising (beyond formal theory of mind) not only makes it possible to parse complex utterances in speech (Oesch and Dunbar, 2017), but also limits the number of individuals whose minds we can monitor simultaneously (Stiller and Dunbar, 2007; Powell et al., 2012; Krems et al., 2016) as well as directly affecting the complexity of the stories we can enjoy (Carney et al., 2014). Without the capacity to mentalise, we would be unable to distinguish between the speaker and the mental states of the characters they describe or recognise that an actor is representing a fictional character rather than speaking their own thoughts. Without this ability, we are likely to assume that the action on the stage is real and might be tempted to leave our seats to intervene.

**Table 1:** Questions completed by participants after watching each play excerpt.

A. Identification (based on Tal-Or and Cohen, 2010):
1. I think I understand [name of character] well
2. I understood the events in the scene the way [name of character] understood them
3. While viewing, I felt like [name of character] felt
4. While watching, I could really ‘get inside’ [name of character]‘s head
5. I tend to understand why [name of character] did what [he/she] did
B. Moral approval:
6. I approve of [name of character]‘s behaviour
C. Attribution (from the IPSAQ scale of Kinderman and Bentall, 1996):
*Would you say [name of character] behaved this way because this was:*
7. Something about [name of character]?
8. Something about the other characters?
9. Something about the situation (circumstances or chance)?

*All answers were on a Likert scale (1=strongly disagree; 7=strongly agree). Question 6 (Moral Approval) was converted to a scale of−3 (strongly disapprove) to+3 (strongly approve), centred on 0 (neither approve nor disapprove) so as to make the analyses more intuitive*.

Becoming engaged in (or transported by) a story or play reflects the extent to which we invest emotional as well as mental effort in decoding the text (Green et al., 2004; Wirth, 2006). When we identify with a character, we care deeply about the character and worry about what will happen to them (Green et al., 2004). Moreover, the degree to which we identify with a character in a story can subsequently affect our opinions about the story or drama (Green, 2005). Tal-Or and Cohen (2010) drew attention to the fact that identification and transportation (both of which are known to affect enjoyment) may often be confounded in many studies; they concluded, on the basis of careful experimental manipulation, that these are in fact independent dimensions of the sense of enjoyment that audiences gain when reading or watching fiction. Identification can thus be an important dimension of an audience’s engagement with a play.

In addition to these more conventional aspects of our ability to engage with the characters in a story, we also here consider two additional dimensions: moral approval and attributional style. Though rarely given as much attention, an individual’s moral attitude towards a character is likely to be important in how they engage with a story. Their view of whether a character is acting morally or immorally (irrespective of the standard against which this might be judged) might well colour both their willingness to identify with, or warm to, a character as well as their ability to make appropriate attributions about the character’s motives. While there is an extensive literature on the psychological bases and ontogeny of moral attitudes (e.g. Kagan and Lamb, 1987; Baird and Astington, 2004), and much commentary by literary scholars has focused on the moral status of characters’ behaviour (e.g. among many others, Baines, 1980; Zamir, 2007; Laam, 2010), the role of moral approval/disapproval as an audience response to characters has not usually been considered a variable of interest in experimental studies of fiction.

Attributional style, on the other hand, is the tendency to explain personally significant events in particular ways. This is sometimes seen as reflecting an individual’s natural psychological style (they blame other people or circumstances for the disasters that befall them rather than taking the blame themselves) but equally provides a useful way of understanding other people’s behaviour (someone acted as they did because that was just their personality or because of the circumstances they found themselves in). Central to this, and of particular relevance for the present study, is the concept of ‘locus of control’ (Rotter, 1966), with its emphasis on the way external versus internal factors are viewed as influencing events. Attributional style has been applied to a wide range of psychological disorders (Abramson et al., 1978; Bentall et al., 1994; Buchanan and Seligman, 1995) as well as the behaviour of normal individuals in a variety of contexts (Kinderman et al., 1998) and has commonly been interpreted as underlying pessimistic (especially reflected in learned helplessness or hopelessness) versus optimistic attitudes to life. While attributional style has been viewed as an essentially endogenous trait, the concept of locus of control lends itself describing the behaviour of third parties, and we here use it as a framework to ask how subjects ascribe locus of control to a character in a play and whether this in turn influences their engagement with the character.

There has been a longstanding interest in how we infer the intentions of characters in fictional literature (Ross, 1977; Pollard-Gott, 1993; Culpeper, 1996). Tal-Or and Papirman (2007), for example, found that subjects viewing short movie clips were more likely to commit the Fundamental Attribution Error (FAE, the tendency to automatically attribute behaviour to internal motivations rather than external circumstances) by attributing behaviour to the actor’s personality than to the fact that the actor was simply following a script. Moreover, the level of transportation in response to the clip was significantly correlated with the magnitude of the FAE. Attribution thus has some potential to offer insights into judgements about characters. Attributional judgements might well affect an individual’s empathy with a character, for example, and hence their willingness to engage (or identify) with the character. We used Kinderman and Bentall’s (1996) IPSAQ, in which attribution involves three mutually exclusive causal loci: dispositional, external personal and external situational. These ascribe an individual’s actions to their own intrinsic psychological make-up or personality (dispositional), the influence of other people on the individual (external personal: hereafter, simply ‘external’) or circumstances largely beyond the individual’s control (external situational: hereafter, ‘situational’). We ask whether subjects identify more with a character in a play if they feel that the character’s locus of control is beyond his/her control (e.g. due to the circumstances they find themselves in or to the behaviour of other characters).

Experimental studies of fiction have largely involved written stimuli (selections from novels or specially written fictional or pseudo-factual accounts) or have focussed on the influence of film or TV (Drabman and Thomas, 1975; Huesmann et al., 2003; Huesmann and Eron, 2013). Here, we use live-performed scenes from staged drama: the opening scenes from Shakespeare’s tragedy *King Lear* (1607) and the opening scenes from Sophocles’ tragedy *Antigone* (second half of the fifth century BC). We chose these plays for two reasons. First, tragedies are most likely to arouse deep emotional responses in some (though not necessarily all) of those who watch them (Dunbar et al., 2016), thus maximising the likelihood of differential responses. Second, the two plays are separated by two millennia and are the product of very different cultural and theatrical contexts, thereby providing some scope for addressing the question of how universal any responses might be. Finally, an important component of how well storytelling (including dramatic storytelling) works its magic lies in the way the story has been conceived and how it is told. To address this, we presented the plays in two very different ways – different in interpretation, costume and set while using the same text and the same actors.

It is a truism of performance studies that one never sees *the* play; one sees only *one version* of the play. The actor Tony Church (1989) who played Polonius at the Royal Shakespeare Company in 1965 and 1980 describes the very different character he played in Peter Hall’s political 1965 production (Claudius running a spy-state, with Polonius a Machiavellian courtier) versus John Barton’s 1980 domestic version with its focus on families (Polonius as father; Brockbank, 1989). We commissioned two radically different interpretations of *Antigone* and *King Lear*, using the identical text for both interpretations and the same actors and director. In one version, the characters were presented sympathetically; in the other, the characterisations were reversed so that the good characters now became the bad ones and vice versa (reversed characterisation). This allowed us to assess whether the dramatist’s storyline (which remained unchanged) or the way the story was told (the input of the actors and the director) had a greater impact on the audience’s response.

We ask three specific questions with respect to how subjects view the behaviour of the characters in the play. We ask first whether audience members differentiate in similar ways between characters on the dependent variables (i.e. Identification rating, Moral Approval and Attribution rating), and whether this is influenced by their familiarity with the plays or the different interpretive staging of the plays. We then ask whether the extent to which participants identify with a character correlates with their moral approval of that character’s behaviour. Third, we ask whether participants are more likely to morally approve of a character’s behaviour if they view that behaviour as being determined by external circumstances (external or situational attribution) rather than the character’s own psychological disposition (dispositional attribution).

## Materials and Methods

### Participants

Eighty-three participants (33 male, 46 female; 4 declined to specify gender; mean age 26.8±12.7years, range 18–71) were recruited by advertisements and attended one of two performances, for which they were paid £5. Of the participants, 62 (76%) were currently enrolled as university students, of whom 16 (19% of all participants) were studying English and 23 (28%) were studying Classics and so might be expected to be especially familiar with one or both of the plays. Prior to watching the performances, participants completed a general background questionnaire (demographic and educational background). They then watched the two scenes (c.20min performance time each), after which they completed a questionnaire on their responses to the characters at the end of each version. The experimental design was between-subjects with each audience group watching either the conventional or the reversed characterisation versions (but never both).

The study was approved by the University of Oxford’s Central University Research Ethics Committee.

### Stimuli and Presentation

Participants watched live performances of the opening two scenes of Shakespeare’s *King Lear* and the opening three scenes of Sophocles’ *Antigone* (in English translation). Of these, five of the characters in the *Lear* excerpt have significant speaking parts (Lear, Cordelia, Goneril, Edmund and Gloucester), and three (Antigone, Creon and Ismene) have significant speaking parts in *Antigone.* (We did not include lines delivered by the chorus in *Antigone*.) All the actors were members of the university or college dramatic societies and were used to public performance to a high standard; the directors were student directors with considerable experience directing plays for public performance. The plays had been fully rehearsed and the actors and directors were each paid £20. The performances were given on stage, with the participants seated in an auditorium. Subjects watched *Antigone* first and *Lear* second.

Each play was presented in two radically different versions (conventional characterisation and reversed characterisation) in order to determine whether the neutral text (which stayed the same) or the performance (which was presented differently) plays the more important role in influencing audience engagement. Half the participants watched the conventional characterisation and half watched the reversed characterisation of both plays. One director was responsible for the two versions of *Lear* and another director was responsible for the two versions of *Antigone*. The same set of actors and directors performed both versions of each play. The conventional version of *Lear* (sympathetic to Lear and Cordelia) was presented in mediaeval dress with warm palettes and rich fabrics, opening on a relaxed and sociable banquet. This is a world in which authority is humanised, parental dignity wounded, and Lear’s tearful outrage a painful and unexpected climax; Edmund is scheming and resentful, and his father Gloucester out of his depth. In the reversed characterisation version, the script was identical but the way the characters were portrayed was reversed: the mild and anguished ones were presented as grasping and aggressive, while the scheming and hostile ones were presented as mild and confused. In this version, *Lear* was staged in modern dress as a board meeting of a family firm at which the eponymous patriarch divides the family empire. Lear was portrayed as a psychopathic, focussed businessman concerned to ensure the future of his business empire; Cordelia accepts, yet at the same time resents, her position as favourite, pandering to her father with disdain; her two sisters are vulnerable and strained – out of their depth in the boardroom politics between their father and Cordelia – while Gloucester is physically aggressive and Edmund marginalised and denigrated. In the two versions of *Antigone*, the eponymous heroine was first staged as an ethically principled absolutist (the conventional portrayal) and in the second as a morally rebellious teenager. In the first version, Creon is tyrannically non-negotiable, a moral traffic warden for whom a rule is a rule, whereas in the second version he is a humane ruler placed in an impossible position, all handwringing anguish and indecision. The two performances of each play were dramatically very different.

Participants were not given any information about the task or asked to focus on anything in particular but were merely told that they would be asked to rate their enjoyment of the two plays they were about to see. At the end of each performance, participants completed a questionnaire which asked them to rate, for each main character separately (a) how much they identified with the character [five questions, each on a 1–7 Likert scale, using the instrument from Tal-Or and Cohen, 2010 based on Cohen (2001); Identification questions], (b) whether they approved of the character’s behaviour (Moral Approval question: responses on a Likert scale, converted to −3=strongly disapprove, +3=strongly approve, with 0 as midpoint), and (c) using the Kinderman and Bentall (1996) IPSAQ instrument, whether they thought the character’s behaviour could be attributed to the character’s intrinsic nature (Disposition), the behaviour of the other characters (External) or something about the situation that the character found him/herself in (Situation; also on a 1–7 Likert scale; Attribution questions) (Table 1). For *Lear*, participants were asked these questions about the five key characters in the excerpts, namely, Lear, Cordelia, Goneril, Gloucester and Edmund; for *Antigone*, they were asked about the three main characters, namely, Antigone, Ismene and Creon. For analysis, the Likert scale scores for each of the five Identification questions were averaged to give a mean overall score for this variable.

Participants were also asked to state how often they had either read or seen each play (on a 4-point scale: never, once, twice, three or more times). The reading and viewing scores were highly correlated within plays (Spearman r_S_>0.447, *p*<0.001), but, perhaps not too surprisingly, only very weakly correlated between plays (0.096≤r_S_≤0.334). Of the 83 participants, 30 had never read and 35 had never seen a performance of *Lear*, and 25 had never read and 48 never seen *Antigone*. Only 21 had seen *Lear* more than once and only 10 had seen *Antigone* more than once. For the analyses, the scores for reading and viewing for each play were added together to give a Familiarity score for that play (which could therefore range from 0 to 6; Supplementary Material Data Sheet 1).

### Statistical Analyses

Since most variables are not normally distributed, we use non-parametric statistical tests. However, for comparisons across characters and for multivariate analyses, we use analysis of variance as this is robust to departures from normality. In each case, effect sizes are given in a form appropriate to the particular statistical test used for an analysis, but only for significant effects.

All statistical analyses were executed in SPSS v.27.

## Results

Descriptives for variables for each character in each play are given in Table 2.

**Table 2:** Descriptives for conventional characterisation^*^ performance.

Play	Character	Identification (mean)	Moral approval^†^	Disposition	Attribution scale		Familiarity with play
					External	Situation	
*Antigone*	Antigone	5.3 ± 1.2	1.0 ± 1.5	6.2 ± 1.2	5.3 ± 1.2	4.8 ± 1.8	1.92 ± 1.7
	Ismene	4.1 ± 1.0	0.1 ± 1.3	5.3 ± 1.2	5.4 ± 1.3	5.4 ± 1.2	
	Creon	4.2 ± 1.0	−1.6 ± 1.4	5.9 ± 1.3	5.0 ± 1.5	4.6 ± 1.6	
*Lear*	Lear	3.9 ± 1.1	−2.2 ± 1.1	6.5 ± 0.7	3.7 ± 1.8	4.4 ± 1.4	2.31 ± 2.1
	Cordelia	5.3 ± 0.7	1.1 ± 1.2	6.2 ± 1.0	4.6 ± 1.5	4.7 ± 1.4	
	Goneril	4.2 ± 1.1	−0.9 ± 1.3	5.6 ± 1.2	5.3 ± 1.5	5.1 ± 1.4	
	Edmund	4.6 ± 1.0	−1.5 ± 1.3	6.1 ± 0.8	5.8 ± 1.6	4.5 ± 1.7	
	Gloucester	4.7 ± 0.9	−0.2 ± 1.3	5.4 ± 1.3	4.9 ± 1.5	5.3 ± 1.5	

*All values are Likert scores on a 1–7 scale*.

* For the reverse characterisation performances, see

Figure 1.

† *Likert scores on this trait were converted so as to range from−3 (strongly disapprove) to+3 (strongly approve), with 0 as the midpoint*.

**Figure 1:**
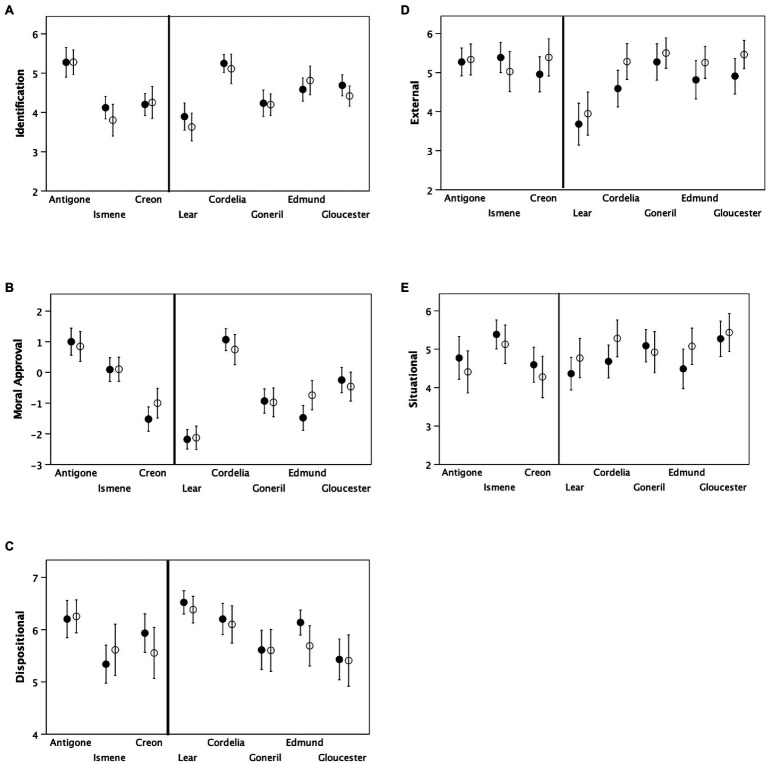
Mean (±95% CI) for the five variables for each of the main characters in the two plays for (A) composite score on the Tal-Or and Cohen (2010) Identification scale, (B) Moral Approval ratings and (C-E) scores on the three dimensions of the Kinderman and Bentall (1996) Attribution scale. Filled symbols: conventional characterisation; unfilled symbols: reversed characterisation.

We first test whether participants consistently differentiate between characters and whether these judgements are influenced by either their familiarity with the plays or how the play was staged. Table 3 indicates that participants rated the characters in a very consistent way: in each case, Cronbach’s alpha for the mean Identification score indicates acceptable levels of agreement across audience members. Figure 1 plots ratings for each character under the two performance conditions. We ran MANOVAs to determine whether the ratings on the five outcome variables (mean Identification rating, Moral Approval and the three IPSAQ attributional scales) were influenced by character, familiarity with the texts or staging (how the play was performed; Table 4). Irrespective of how the play was staged, there was a significant effect of character in every case (except Situational Attribution in *Antigone*), with no effect due to familiarity with the material (again with one exception). With four exceptions, there were no consistent effects due to staging. The exceptions were that the reversed staging resulted in consistently higher ratings for External and Situational Attribution in *Lear*; in addition, there were significant character*staging interaction effects for Dispositional Attribution in *Antigone* (where the ratings for Ismene and Creon reversed) and Moral Judgement in *Lear* (where the ratings for Cordelia and Edmund reversed). Dropping Familiarity increases the significance levels somewhat on all tests but does not change the overall pattern of results [except for the effect of Performance on Identification ratings in *Lear*, which changes from being marginally non-significant (*p*=0.084) to being significant (*p*=0.027)].

**Table 3:** Cronbach’s alpha scores on summed responses to the five identification questions across participants for the eight main characters in the two plays.

Character	Cronbach’s α	Rating^*^
Antigone	0.865	Good
Ismene	0.827	Good
Creon	0.737	Acceptable
Lear	0.713	Acceptable
Cordelia	0.823	Good
Goneril	0.772	Acceptable
Edmund	0.796	Acceptable
Gloucester	0.667	Questionable

* Rating given by

George and Mallery (2003).

**Table 4:** MANOVAs comparing ratings across characters and performances (conventional versus reversed characterisation) for each play, with Familiarity as a covariate.

												^*^Attribution scale					
Play	Factor	df	Identification			Moral approval			Dispositional			External			Situational			F	p	*f* ^†^	F	p	*f*	F	p	*f*	F	p	*f*	F	p	*f*
*Antigone*																	
Character		2,224	**40.3**	**<0.001**	**0.58**	**55.4**	**<0.001**	**0.69**	**5.9**	**0.003**	**0.21**	**5.4**	**0.005**	**0.20**	0.4	0.693	
Staging		1,224	0.3	0.607		0.2	0.649		0.4	0.507		0.1	0.785		0.8	0.369	
Interaction^§^		3,22	0.1	0.895		1.7	0.186		**3.9**	**0.021**	**0.16**	0.1	0.910		0.9	0.406	
Familiarity		1,224	0.2	0.644		1.9	0.173		**5.8**	**0.017**	**0.14**	2.4	0.126		0.6	0.438	
*Lear*																	
Character		4,377	**22.8**	**<0.001**	**0.47**	**55.5**	**<0.001**	**0.75**	**10.3**	**<0.001**	**0.31**	**2.5**	**0.039**	**0.13**	**12.7**	**<0.001**	**0.34**
Staging		1,377	3.0	0.084		1.5	0.229		1.1	0.286		**6.5**	**0.11**	**0.12**	**5.3**	**0.021**	**0.11**
Interaction^§^		5,377	0.8	0.516		**3.2**	**0.014**	**0.15**	1.1	0.336		1.6	0.173		0.1	0.970	
Familiarity		1,377	0.0	0.957		0.2	0.681		1.0	0.307		2.8	0.096		2.1	0.144	

*Significant effects are highlighted in bold*.

† *Effect size (Cohen’s f_effect_), given for significant effects only.*

§ *Character.*

* *Staging interaction.*

The patterns of identification in the conventional, sympathetic performances were exactly as one would expect: participants identified more readily with Cordelia than with Lear, and morally approved of Cordelia’s behaviour more than anyone else’s, and they similarly identified more with Antigone than with Creon or Ismene, and morally approved of her behaviour more. That there should be some (albeit limited) effect of staging on ratings for Ismene, Creon, Cordelia and Edmund is not too surprising, since these are the characters that, in professional productions, tend to arouse the most extreme responses in audiences. Antigone, caught in an extremely difficult situation, is rated positively no matter how she is presented, and Lear always receives low ratings (perhaps because, in these opening scenes and in the absence of the pity he might arouse later in the play, he is seen as instigating division within the family irrespective of how he might be doing this).

It is worth noting that the moral approval ratings for lesser characters did exhibit some response to performance type: at least in *Lear*, there was a striking tendency for most ratings of Edmund, Goneril and Gloucester to converge in response to the reversed characterisation performance, although they all remained in the mid-ground between Lear and Cordelia (Figure 1B). Notably, the main characters did not exhibit such shifts in audience perception, perhaps because these characters are more tightly drawn by the text with less scope for alternative interpretations or are characters that, for dramatic effect in the compressed timescale of a play, are more exaggerated in order to push the audience into entering more firmly into the action of the play as conceived by the playwright.

So far, we have established two key points: audiences discriminate reliably between characters and their ratings are, by and large, unaffected by how the play is staged or by their familiarity with the play(s). The latter finding implies that the words that the dramatist places in the mouths of the characters are more important in defining our sense of engagement with them as individuals than how actors might choose to portray them.

To examine our second question (the extent to which the rating variables correlate with each other), we pooled the two performances for each play in the light of the fact the staging has very little effect on ratings. We first consider how consistently participants rated their moral approval of the main characters. In all cases except Ismene, they typically came down firmly on one side or other as to whether or not they morally approved of a character’s actions (Table 5). The average proportion of scores that are on the minority side of the midpoint is just 17.7% across the eight characters. Thus, with the exception of Ismene, the scores are typically truncated at the midpoint (the point of ambivalence): in other words, participants took a consistent view on each character. Within this constraint, Moral Approval ratings correlated positively with Identification score for six of the eight characters in the two plays, even with Bonferroni correction (Table 6). The exceptions were Goneril and Edmund, and even these yielded positive (albeit non-significant) correlations. In other words, participants morally approved of a character’s behaviour roughly in proportion to the extent to which they identified with that character.

**Table 5:** Distribution of moral approval ratings for characters (two performances pooled).

Character	Mean rating^*^ ± SD	% minority rating (below or above 0)	N^†^	t	Effect size^§^	p
Antigone	0.92±1.51	15.9	82	5.53	0.61	<0.001
Ismene	0.10±1.24	27.7	83	0.71	0.08	0.479
Creon	−1.33±1.42	31.7	82	−8.53	0.94	<0.001
Lear	2.17±1.11	2.4	82	−17.75	1.95	<0.001
Cordelia	0.92±1.35	14.5	83	6.20	0.68	<0.001
Goneril	−0.95±1.35	13.4	82	−6.38	0.70	<0.001
Edmund	−1.13±1.44	9.8	83	−7.18	0.79	<0.001
Gloucester	−0.36±1.42	26.8	82	−2.32	0.26	0.023

* *Rating scale is−3 to+3, with midpoint at 0*.

† *One participant did not answer all questions*.

§ *Cohen’s d*.

**Table 6:** Correlations between mean identification and moral approval ratings for each character.

Play	Character	Spearman r	Effect size (r^2^)	N^§^	*p*
*Antigone*	Antigone	0.662	0.44	82	<0.001
	Ismene	0.439	0.19	83	<0.001
	Creon	0.296	0.09	82	0.007
*Lear*	Lear	0.350	0.13	82	0.001
	Cordelia	0.609	0.37	83	<0.001
	Goneril	0.177	0.03	82	0.112
	Gloucester	0.445	0.20	83	<0.001
	Edmund	0.188	0.04	83	0.089

§ *Sample sizes vary slightly because one participant did not answer all questions*.

Our third question concerns the extent to which moral approval of the individual characters’ behaviour correlates with the participants’ perception of what was to blame for how the characters behaved. In *Antigone*, the behaviour of both Creon and Antigone was viewed as largely due to their own dispositions, whereas that of Ismene owed its origin more to the situation in which she found herself. In *Lear*, the behaviour of Lear and Cordelia is attributed to their dispositions, but with some effect due to external influences in the case of Cordelia. Edmund’s behaviour is also seen as reflecting his disposition, but in many respects, participants were more ambivalent about him. Goneril and Gloucester were both seen as victims of circumstance. Moral Approval ratings did not correlate significantly with any of the Attribution ratings for any of the eight characters (Spearman correlations, all *p*>0.05) with just two exceptions (Antigone for External Attribution, *p*=0.007; Creon for Dispositional Attribution, *p*<0.001). This suggests that, generally speaking, moral approval was given independently of the explanation that audience members offered for a character’s behaviour.

## Discussion

We have shown that members of an audience reliably differentiate between characters in two canonical tragedies from very different historical periods in terms of their ability to identify with characters (a capacity that we equate functionally with mentalising), their moral approval of the character’s behaviour and their attribution of the psychological causation of a character’s behaviour. However, contrary to expectation, there was no overall performance-by-character interaction for these variables, suggesting that directorial style had less influence on the audience’s ratings for the set of characters in a play than the text itself allows, even when controlling for prior familiarity with the plays. In this respect, drama and other forms of storytelling may differ from everyday social interactions: in real-life contexts, how something is said seems to be more important than what is said, at least as far as identifying the quality of the relationship between two speakers is concerned (Dunbar et al., 2021). This may be because storytellers need to craft their characters more precisely in the compressed temporal context of a play or story. Secondly, we found that, irrespective of performance, the audience’s identification with a character correlated with the extent to which they morally approved of the character’s actions. However, neither of these correlated with their attribution of the psychological causes of individual characters’ behaviour, notwithstanding the fact that they consistently distinguished between attributional styles in respect of the various characters. This suggests that attributional style was more dependent on the character as drawn by the playwright than an audience member’s ability to identify with a character or the moral stance that they took in respect of the character.

Our results, thus, suggest that the broad findings that have been reported for narrative literature in respect of identification and engagement (‘transportation’ in the terminology of Green and Brock, 2000) also apply to live dramatic performances, indicating that differences between modes of storytelling (readerly versus performance-based) may be considerably less than the similarities (see also Green and Brock, 2000; Sherry, 2004). The fact that our subjects exhibited a strong correlation between identification with a character and the level of moral approval for that character confirms the earlier finding that readers of fiction who become highly ‘transported’ are generally more positive towards characters (Green and Brock, 2000). Identification and transportation in this sense are not, of course, identical concepts, but transportation does seem to be important for readers to be able to develop a sense of identification with a character (Green et al., 2004).

Psychological studies of audience engagement have tended to focus on the concept of identification as one of the key processes underlying ‘transportation’ (or ‘narrative transportation’; Gerrig, 1993; Green and Brock, 2000) whereby readers become engaged with (or engrossed by) a text through relating to a character. Van Laer et al. (2014) suggest that narrative transportation is the outcome of two key processes (the individual being able to empathise with the characters and the story plot activating his or her imagination) that between them lead to a state of suspended reality. The result is a psychological separation between the fictional world and the real world within which the reader is actually situated. Without being able to keep these two worlds mentally separate, the reader would treat the fictional world as real, a problem that is likely to be magnified with staged drama because the action on the stage is performed by real people and looks like (and hence could be mistaken for) real life (see Bullough, 1912; Elam, 2002).

Nonetheless, an important conclusion from our results is that, despite becoming engaged with a character, audience members are able to maintain sufficient distance to take an independent view of the psychological causes of the characters’ behaviour. It is this capacity to step back from the immediacy of the present world to consider an alternative parallel world (whether fictional or a real world elsewhere in space or time) that allows us to hold back and remain in our seats rather than intervene on behalf of the victim in a play. Cognitively, this is no small feat: it depends on high-order mentalising capabilities that are neurophysiologically expensive (Lewis et al., 2017) and involve complex extended neural networks (Powell et al., 2010; Lewis et al., 2011; Brown, 2020). Young children, especially those too young to have developed theory of mind, typically see characters as real and only come to recognise the difference between fiction and reality as they develop high-order mentalising skills (Morison and Gardner, 1978; Harris et al., 1991; Chandler, 1997). This finding would seem to have implications for everyday real-world psychology where we may often face a dilemma in which our relationship with (or attachment to) a particular individual (notably close friends and family) conflicts with our ability to provide rational psychological explanations for their behaviour. That we are able to separate out these two dimensions may be important for our capacity to provide balanced, sensible advice to those with whom we are emotionally bound – something that may be crucial in maintaining the integrity and cohesion of a social community.

Mentalising is also important from the storyteller’s perspective. To be able to tell an engaging story, the storyteller needs to construct both the characterisation and the unfolding story in such a way as to guide the audience’s beliefs and engagement. Engagement may be lost when the narrative becomes implausible or too complex (Tal-Or and Cohen, 2010). Storytellers must also constrain their construction of a plot to fit the audience’s psychological competences such that these are not over-taxed (Dunbar, 2005, 2017; Zunshine, 2006). Analysis of the structure of Shakespeare’s plays, for example, demonstrates both that the number of speaking parts in individual scenes approximates very closely to the size of everyday conversation groups and that the structure of networks based on co-presence in the same scene exhibits classic ‘small world’ patterns of the kind found in natural social networks (Stiller et al., 2004). This is true even of contemporary hyper-link cinema (Krems and Dunbar, 2013), despite the fact that this particular genre (which includes films, such as *Crash*, *Babel* and *Love Actually*) explicitly attempts to break through these everyday limitations by linking the lives of individuals whose actions are dissociated in time and space. While these structural components to a story are important in order to avoid overburdening audience psychology, the key to storytelling nonetheless lies ultimately in the psychological mechanisms that draw the audience in.

Disposition theory (Zillmann and Bryant, 1975; Raney, 2004; Weber et al., 2008), which argues that audience enjoyment is high when characters who are disliked experience negative outcomes (and vice versa), might be an alternative explanation for our findings. Disposition theory identifies empathy and similarity/dissimilarity (i.e. in-group bias) as important psychological components, but ultimately a sense of (social) justice is thought to play a central role (Raney and Bryant, 2002). Raney and Bryant (2002) argue that enjoyment of drama (film) is the intersection of judgements made about characters (disposition formation) and judgements made about justice (a moral view). In an empirical test of this, they found that these two ratings were independent and that the two together significantly predicted enjoyment of short film clips (accounting for ~23% of the variance between them). While this hypothesis was explicitly developed for crime drama as a genre, the general approach can easily be generalised to other forms of drama (e.g. soap operas: Weber et al., 2008). To the extent that moral approval is one of the core elements in disposition theory (Raney, 2004; Weber et al., 2008; Zillmann and Bryant, 1975), our results offer some support for this proposal.

Other potentially important traits that we did not investigate, such as experience-taking (the ability to enter into the experiences of a character: Kaufman and Libby, 2012) and empathy and sympathy (Goldstein and Winner, 2012), are also likely to play an important role in individual differences in the ability to become immersed in fiction. More importantly, perhaps, the storyteller’s ability to trigger these mechanisms through choice of words or narrative structure may play a crucial role in eliciting a response from an audience (Kaufman and Libby, 2012). Kaufman and Libby (2012) have suggested that ‘experience-taking’ may be an important component of engagement because it allows an individual to transcend the self-other boundary. In a series of experimental studies, they showed that high self-concept accessibility (the capacity to reflect on the causes of one’s own behaviour) blocks engagement in fictional narratives (apparently because individuals are unable to enter into the world of the depicted character), whereas in-group cues narrated in the first person have a positive effect on engagement. Similarly, Goldstein and Winner (2012) used live stage performances to examine the extent to which cognitive empathy (understanding another’s emotions), emotional empathy (feeling another’s emotions) and personal distress (experiencing a negative emotional reaction to another’s plight) predict sympathy for characters in two staged plays. They found striking sex differences, with level of sympathy best predicted by emotional empathy in men but by cognitive empathy in women.

It is notable that psychologists have largely failed to engage with the fictional world of literature despite the fact that it offers an opportunity to explore the psychology of a mental world that not only plays a significant part of everyday life but may also be one that reflects psychological processes that are fundamental to everyday human behaviour (Goldstein and Bloom, 2011). In this respect, there have been surprisingly few experiments that have used audiences with live drama to explore appreciation of, and attitudes towards, fiction. Although working with audiences at live performances is inevitably fraught with difficulties, not least because confounds are less easily controlled than they are in the laboratory, we can have some satisfaction in how well this particular experiment worked. Despite many potential problems, the results we obtained seem to be robust and, where they mirror results from previous laboratory experiments, reliable. They also appear to be consistent across plays that are separated both by almost two millennia in time and by cultural background. This gives us some confidence in the generality of the findings we report, such as the fact that audiences are able to disengage their level of identification and moral approval for a character from the psychological explanations they are prepared to give for the character’s behaviour; the consistency of these ascriptions across plays not only from very different theatrical traditions (and historical periods) but also interpreted/performed in very different ways is telling. The explicitly cognitive aspects of our capacity to cope with storytelling and the theatre have yet to be explored in any detail (but see Carney et al., 2014), yet may provide important insights into how we cope with everyday real-world social interactions (including, perhaps, our ability to engage with virtual mental representations of real but absent individuals) as well as offering a better understanding of why, at a psychological level, fiction works for us.

## Conclusion

Our results suggest three main conclusions. One is that the text, as crafted by the playwright, takes precedence over both the director’s influence and the way the actors present the characters on stage. This may reflect our two particular dramatists’ ability to draw characters so finely that the audience is offered little leeway in how to interpret them, something that may be particularly important for those characters whose behaviour is central to the plot as the dramatist conceives it. It could be that less skilled storytellers are not able to impose their characterisations on their audiences so effectively. More detailed experimental analysis would be required to confirm this, but it offers a possible psychological basis for explaining the differences between successful and unsuccessful storytellers.

Second, the level of moral approval of a character’s behaviour was highly correlated with the participants’ ability (or willingness) to identify with the character and the particular dilemma that the character faced (see also Green and Brock, 2000). Causal direction remains to be determined here and would clearly merit more detailed experimental study. Third, that said, audiences clearly differentiated between identification with a character and moral approval of their actions, on the one hand, and the attributional explanations they offered for the character’s behaviour, on the other: they seemed able to take a consistent view of the psychological causes of a character’s actions that was independent of whether or not they approved of how the character had behaved. This cognitively demanding, yet largely unresearched, mechanism that allows us to hold contradictory views of someone in mind at the same time probably plays an equally important role in everyday life by allowing us to engage mentally with people who are not physically present.

## Data Availability Statement

The original contributions presented in the study are included in the article/Supplementary Material, and further inquiries can be directed to the corresponding author.

## Ethics Statement

The studies involving human participants were reviewed and approved by the Central University Research Ethics Committee (CUREC), University of Oxford. The patients/participants provided their written informed consent to participate in this study.

## Author Contributions

BT, LM, FB, and RD conceived the project, designed and carried out the experiments, analysed the data, and wrote the manuscript. All authors contributed to the article and approved the submitted version.

## Funding

The research was funded by the John Fell Fund, and the analysis and writing up were funded by the Calleva Research Centre, Magdalen College, Oxford.

## Conflict of Interest

The authors declare that the research was conducted in the absence of any commercial or financial relationships that could be construed as a potential conflict of interest.

## Publisher’s Note

All claims expressed in this article are solely those of the authors and do not necessarily represent those of their affiliated organizations, or those of the publisher, the editors and the reviewers. Any product that may be evaluated in this article, or claim that may be made by its manufacturer, is not guaranteed or endorsed by the publisher.
